# Design of dendrimeric structures to treat allergic disorders

**DOI:** 10.1186/2045-7022-3-S3-P81

**Published:** 2013-07-25

**Authors:** AB Blazquez, A Aranda, A Mascaraque, MJ Torres, C Mayorga, J Rojo, M Blanca

**Affiliations:** 1 Research Laboratory, IMABIS Foundation-Carlos Haya Hospital/Bionand, Malaga, Spain; 2 Glycosystems Laboratory, Instituto de Investigaciones Químicas, CSIC, Seville, Spain; 3 Allergy Service, Carlos Haya Hospital, Malaga, Spain

## Background

Polymers containing immunogenic peptides bonded to dendrimeric structures have been developed to be used for vaccines in cancer, infectious diseases, and allergy. Our aim is to design a dendrimeric structure containing Ole e 1 and CpG, in order to modulate an allergic immune response towards Th1, in an experimental model of anaphylaxis.

## Methods

C56BL/6 mice were sensitized by intranasal administration of olive extract+cholera toxin B, for 6 weeks. Then, mice received immunotherapy (IT) treatment by subcutaneous (sc) injection of dendrimer-Ole1±50 mg of CpG, for 8 weeks. Seven days after the IT, mice were challenged with 100 mg of olive extract (ip). Severity of anaphylaxis was measured by drop in body temperature and the humoral response by ELISA.

## Results

Olive sensitized mice treated with the dendrimer-Ole1 without CpG developed a drop in body temperature similar to anaphylactic mice (35.02±1.39 vs 34.48±0.82, respectively), indicating that Ole e 1 within a dendrimeric structure is recognized in vivo. On the other hand, 8 weeks after immunotherapy, mice receiving the dendrimer-Ole1+CpG were significantly protected from the development of systemic anaphylaxis (37.18±1.58 vs 34.48±0.82, p‹0.05, respectively). IgE and IgG2a levels decreased after 1-2 weeks of treatment and remained stable over the time; however IgG1 levels were normal.

## Conclusion

Our results indicate that the dendrimer-Ole1 is recognized in vivo. Furthermore, we show that sc administration of dendrimer-Ole1+CpG protects sensitized mice from the onset of anaphylaxis, and the decrease in IgE levels during the IT treatment may be responsible from that protection.

## Disclosure of interest

None declared.

